# Sex-specific associations between diet quality and mortality in adults with diabetes: findings from NHANES 2001-2018

**DOI:** 10.3389/fnut.2025.1576983

**Published:** 2025-04-16

**Authors:** Zhang Youqi, Yan Meng, Ji Liu, Wu Jianjun, Yang Fan

**Affiliations:** ^1^Department of Cardiology, The Second Affiliated Hospital of Harbin Medical University, Harbin, China; ^2^Key Laboratory of Myocardial Ischemia, Ministry of Education, Harbin, China; ^3^Department of Pathology, The First Affiliated Hospital of Soochow University, Soochow University, Suzhou, China

**Keywords:** Type 2 diabetes, healthy eating index, mediterranean diet, all-cause mortality, cardiovascular mortality, gender-specific

## Abstract

**Objective:**

To investigate the impact of diet on cardiovascular (CV)/all-cause mortality among individuals with diabetes, and to explore whether this relationship changes by gender.

**Methods:**

We collected data from the National Health and Nutrition Examination Survey (NHANES) database pertaining to 5,875 individuals with diabetes (3,068 males and 2,807 females) and used the Healthy Eating Index (HEI), the Alternative Healthy Eating Index (AHEI), and the alternative Mediterranean Diet (aMED) index to assess diet quality. Multivariate Cox models were used to determine the association between dietary quality scores and CV/all-cause mortality, stratified by genders. Dose–response relationships were assessed using the Restricted Cubic Spline (RCS). As a secondary objective, a further analysis was conducted on the connection between CV/all-cause mortality and different dietary components.

**Results:**

During a median 9.25-year follow-up period, we observed 1,488 all-cause deaths, including 486 CV deaths. Sex-stratified analyses revealed that higher diet quality, as indicated by each standard deviation increase in the score, was significantly associated with a reduced risk of cardiovascular mortality in males (*p* < 0.05). No significant associations were observed in females (*p* > 0.05). Among the component scores of the aMED, legume intake was unfavorable for males with diabetes but was remarkably associated with lower CV/all-cause mortality in females.

**Conclusion:**

In the diabetic population, high dietary scores are significantly associated with lower CV/all-cause mortality in males but not in females.

## Introduction

Type 2 diabetes mellitus (T2DM) has emerged as a critical global health challenge, currently affecting approximately 537 million adults, with projections indicating an alarming rise beyond 783 million by 2045 (1, 2). Extensive epidemiological evidence underscores T2DM as a significant independent risk factor contributing to multiple severe chronic conditions (3). Individuals diagnosed with T2DM exhibit a substantially elevated complication burden, notably experiencing a two- to five-fold increased risk of myocardial infarction and a two- to three-fold heightened risk of stroke compared to non-diabetic populations (4–6).

Accumulating evidence highlights lifestyle modifications—particularly dietary optimization-as a cornerstone for effective glycaemic control, demonstrating potential to significantly reduce T2DM progression risk by 30–60% (7, 8). An optimal dietary regimen encompasses diversified food selection, precise portioning of staple foods, and abundant consumption of vegetables, fruits, dairy, and legumes (9). Specific dietary patterns, such as the Mediterranean diet (MED), have consistently shown protective effects on CV mortality (10). Indeed, a pivotal longitudinal trial investigating the Mediterranean dietary pattern over approximately 4.3 years observed a substantial 30% risk reduction for cardiovascular disease (CVD) (11). Additionally, meta-analytic evidence has revealed that higher scores in dietary quality indices—including the alternate Mediterranean diet (aMED), the Alternative Healthy Eating Index (AHEI), and the Healthy Eating Index (HEI)-are inversely associated with the incidence of T2DM and diabetes-related mortality (12–14). However, the differential impact of these dietary indices on CV and all-cause mortality among individuals with diabetes of different genders remains under-explored.

Dietary quality indices, such as HEI, AHEI, and aMED, have been widely used to assess dietary patterns and are consistently associated with both diabetes and cardiovascular risk profiles (15–17). The aim of our study is to employ the National Health and Nutrition Examination Survey (NHANES) dataset to explore how different dietary indices (aMED index, AHEI, HEI) affect CV and all-cause mortality for individuals with diabetes of different genders.

## Materials and methods

### Study population

Using NHANES data from 9 cycles (2001–2018) and a multi-stage clustered probabilistic sampling design, we curated a representative cohort of the American population. The national center for health statistics (NCHS) Research Ethics Review Board approved the NHANES study protocol, and all participants provided oral and written informed consent.

**Figure 1:**
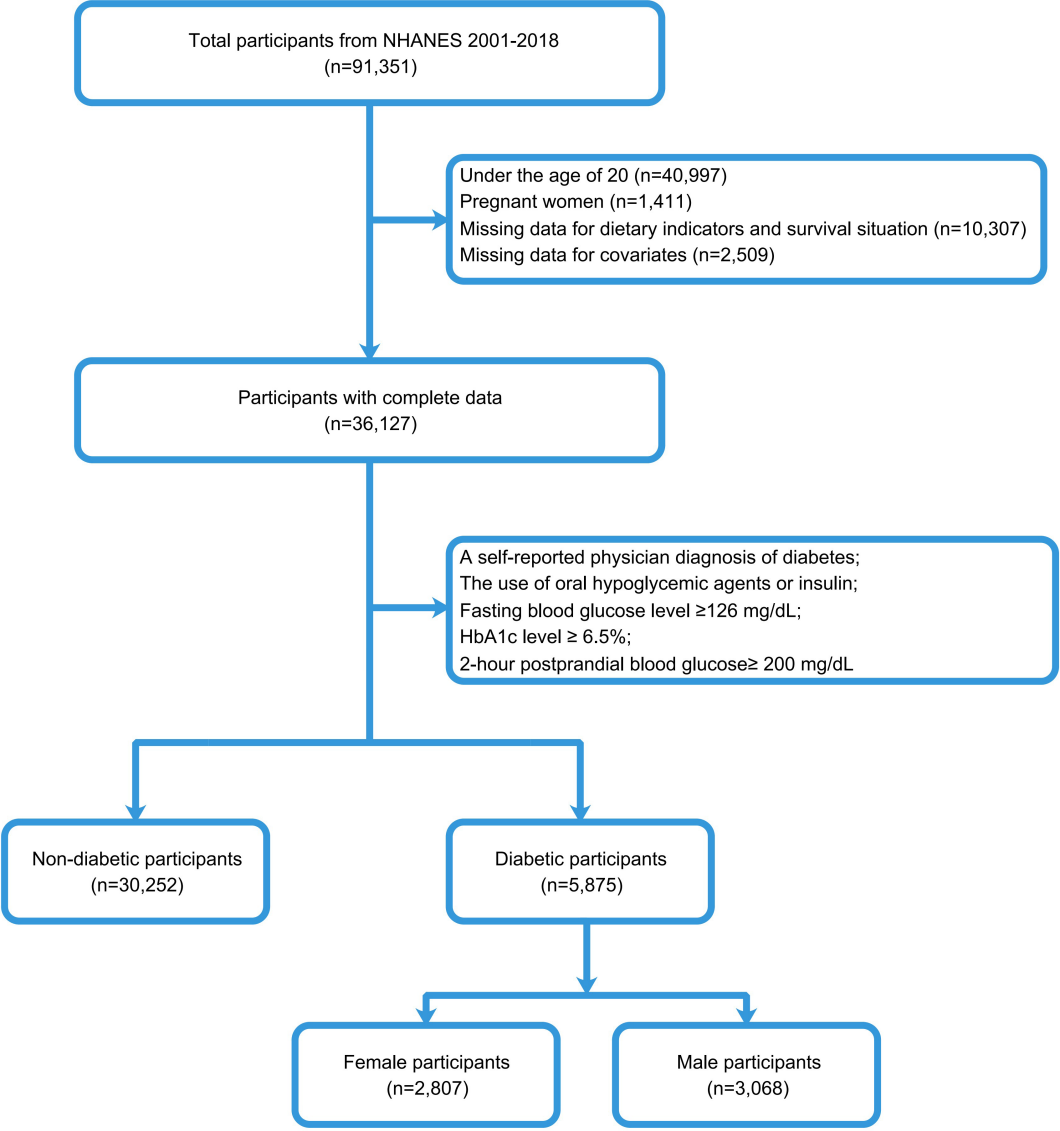
The flow chart illustrates the population selection methodology for this study.

To identify individuals with T2DM, we used the diagnostic criteria of the American Diabetes Association (18). Our study population included adults with diabetes, excluding 40,997 subjects under 20, 1,411 pregnant females, 10,307 individuals without survival status or dietary data, and 2,509 subjects with missing covariates. Finally, according to the diabetes inclusion criteria, 5,875 subjects were included in this study, including 3,068 males and 2,807 females. The entire data selection process was shown in Figure 1.

### Dietary quality scores

24-h recall interviews were used by the NHANES Nutrition Methods Work group to gather dietary data. A total of three dietary indices were analyzed in this study in relation to the CV/all-cause mortality, including: “alternative Mediterranean Diet” (aMED), “Alternative Healthy Eating Index” 2010 version (AHEI-2010) and “Healthy Eating Index” version 2020 (HEI-2020). The scores for each index were calculated according to the methodology reported in the literature, with higher scores indicating better adherence to the dietary pattern.

#### HEI-2020

The HEI-2020 is the latest version, which consists of 13 components categorized as adequate and moderate (19). The adequate components evaluate encouraged foods like fruits, vegetables, whole grains, dairy, protein, and healthy fats, while the moderate components assess limited foods such as saturated fats, refined grains, sodium, and added sugars. HEI-2020 scores range from 0 to 100, with higher scores reflecting higher diet quality.

#### AHEI-2010

The AHEI-2010 consists of 11 dietary items, with 6 components assessing adequacy (vegetables, fruits, whole grains, nuts and legumes, and long-chain n-3 fatty acids; PUFA) and 5 components assessing moderation (sugar-sweetened beverages and fruit juices, red/processed meats, trans fats, sodium, and alcohol) (20). Each component was scored on a 0–10 scale. Because data on trans fatty acids (TFAs) was not collected at baseline in this study (TFAs is one of the 11 items in the AHEI-2010), this item was not included in the scoring.

#### aMED index

The calculation of aMED index was based on the evaluated consumption of fish, whole grains, legumes, nuts, fruits, vegetables (apart from potatoes), red and processed meat, olive oil (the ratio of monounsaturated fatty acids to saturated fatty acids), and alcohol. Participants who consumed more than the median intake in the study cohort were given one point in addition to red/processed meat and alcohol. Additionally, 1 point was given to those who drank moderately (10–25 g/day for males and 5–15 g/day for females) or whose meat intake was below the cohort median. Those who fail to meet these requirements will be given a score of zero (21).

### Covariate assessment

Baseline information was obtained by uniformly trained enumerators using a structured questionnaire to ask participants face-to-face for basic information. Body Mass Index (BMI): The number of kilograms of body weight divided by the square of the number of meters of height. Physical activity level: Participants were categorized as inactive, moderate, or active based on their self-reported physical activity. Smoking status: Participants were defined as smokers and never smokers. Smokers were further divided into ex-smokers and current smokers according to whether they quit smoking. Alcohol consumption: Participants were categorized as non-drinkers and drinkers. Drinkers were further categorized according to the amount of alcohol consumed per day: excessive alcohol consumption (≥ 3 drinks per day for females or ≥ 4 drinks per day for males) and moderate alcohol consumption (≥ 2 drinks per day for females or ≥ 3 drinks per day for males).

### Outcome definition

CV mortality and all-cause mortality were analyzed based on data from December 31 of the 2019 National Death Index. The International Classification of Diseases, Tenth Revision codes accurately determined CV deaths: various heart diseases I00-I09, I11, I13, and ischemic heart disease I20-I51.

### Statistical analysis

We designed our statistical methods according to CDC2022a guidelines to accommodate the complex sampling frame of NHANES. We used Kaplan–Meier curves to evaluate the impact of dietary indices on CV and all-cause mortality in diabetic patients, conducted subgroup analyses to identify special populations, applied multivariate Cox models to determine hazard ratios (HRs) and 95% confidence intervals (CIs), and performed Schoenfeld residual tests to verify the proportional hazard assumption of the Cox model. To ensure result accuracy, we conducted a sensitivity analysis by excluding individuals with CVD to eliminate confounding factors and removing participants who died within the first 2 years to avoid reverse causality. The dose–response relationship was analyzed using Restricted Cubic Spline (RCS) regression in a multivariate framework, with node selection guided by the Akaike Information Criterion (AIC) and linearity assessed through likelihood ratio tests. Finally, we further analyzed the independent correlation between the components of alternative Mediterranean Diet and the CV/all-cause mortality to investigate the magnitude of the contribution of each component to the association between dietary indices and the mortality in diabetic individuals. The model was adjusted for the multivariate in Model 3 above.

R software (version 4.3.2) was employed for all statistical analyses, and a two-sided test was deemed statistically significant if its *p*-value was less than 0.05.

## Results

### Baseline characteristics

The study cohort consisted of 5,875 participants over a median follow-up of 9.25 years (Table 1). Age and gender differences were observed in the dietary indices, where higher age and a greater proportion of females correlated with higher quartiles across all indices. Additionally, HEI, AHEI, and aMED scores were significantly higher among non-smokers, non-drinkers, and those with higher levels of education. Furthermore, lower dietary scores were associated with higher levels of serum HbA1c, TC, FBG, TG and higher TyG values and TyG-BMI values.

**Table 1:** Baseline characteristics of participants with diabetes.

	HEI		AHEI		aMED index	
	Q1	Q4	Q1	Q4	Q1	Q4
	<43.2	≥59.1	<30.1	≥44.7	<5.0	≥6.5
**Patients, n**	1475	1470	1461	1472	827	1822
**Age, years**	60.0 (47.0, 68.0)	66.0(57.0, 74.0)	60.0 (48.0, 69.0)	64.0 (55.0, 73.0)	59.0 (46.0, 68.0)	65.0 (56.0, 74.0)
**Sex/gender, Female**	627 (42.5)	760 (51.7)	664 (45.4)	692 (47.0)	348 (42.1)	925 (50.8)
**Race/ethnicity**						
Non-Hispanic Black	396 (26.8)	310 (21.1)	440 (30.1)	274 (18.6)	232 (28.1)	381 (20.9)
Non-Hispanic White	601 (40.7)	584 (39.7)	560 (38.3)	583 (39.6)	347 (42.0)	711 (39.0)
Mexican American	271 (18.4)	276 (18.8)	234 (16.0)	311 (21.1)	116 (14.0)	391 (21.5)
Other	207 (14.0)	300 (20.4)	227 (15.5)	304 (20.7)	132 (16.0)	339 (18.6)
**Smoking status**						
Current smoking	387 (26.2)	116 (7.9)	376 (25.7)	133 (9.0)	215 (26.0)	153 (8.4)
Former smoking	478 (32.4)	514 (35.0)	454 (31.1)	508 (34.5)	265 (32.0)	637 (35.0)
Never	610 (41.4)	840 (57.1)	631 (43.2)	831 (56.5)	347 (42.0)	1032 (56.6)
**Drinking status**						
Heavy drinking	599 (40.6)	537 (36.5)	583 (39.9)	614 (41.7)	353 (42.7)	684 (37.5)
Low-to-moderate drinking	692 (46.9)	642 (43.7)	660 (45.2)	622 (42.3)	353 (42.7)	803 (44.1)
Never	184 (12.5)	291 (19.8)	218 (14.9)	236 (16.0)	121 (14.6)	335 (18.4)
**Educational attainment**						
College or above	608 (41.2)	694 (47.2)	550 (37.6)	760 (51.6)	329 (39.8)	871 (47.8)
High school or equivalent	347 (23.5)	342 (23.3)	355 (24.3)	305 (20.7)	195 (23.6)	396 (21.7)
Less than high school	520 (35.3)	434 (29.5)	556 (38.1)	407 (27.6)	303 (36.6)	555 (30.5)
**Exercise status**						
Active	66 (4.5)	55 (3.7)	70 (4.8)	51 (3.5)	44 (5.3)	64 (3.5)
Inactive	889 (60.3)	897 (61.0)	911 (62.4)	867 (58.9)	512 (61.9)	1071 (58.8)
Moderate	692 (46.9)	642 (43.7)	660 (45.2)	622 (42.3)	353 (42.7)	803 (44.1)
**Hypertension, yes**	908 (61.6)	1007 (68.5)	908 (62.1)	960 (65.2)	499 (60.3)	1198 (65.8)
**CVD, yes**	348 (23.6)	381 (25.9)	389 (26.6)	307 (20.9)	206 (24.9)	473 (26.0)
**BMI, kg/m** ^**2**^	32.4 (27.9,37.6)	30.1 (26.7,34.6)	31.9 (27.8,37.2)	29.9 (26.4,34.4)	31.9 (27.8, 37.3)	30.2 (26.6,34.7)
**HbA1c, %**	6.9 (6.2, 8.1)	6.8 (6.2, 7.7)	6.8 (6.2, 8.0)	6.8 (6.1, 7.7)	6.9 (6.4, 8.2)	6.8 (6.2, 7.8)
**TC, mg/dL**	187.0 (158.0, 219.0)	182.0 (155.0, 216.0)	189.0 (161.0, 221.0)	183.0 (155.0, 215.0)	190.0 (158.0, 222.0)	183.0 (156.0, 214.0)
**FPG, mg/dL**	130.0 (107.0, 177.0)	126.0 (105.0, 161.0)	128.0 (106.0, 175.0)	126.0 (104.0, 164.0)	131.0 (109.0, 182.0)	127.0 (105.0, 168.0)
**TG, mg/dL**	157.0 (108.0, 238.0)	148.0 (97.0, 217.0)	157.0 (108.0, 234.0)	146.0 (96.0, 212.0)	157.0 (105.0, 241.5)	148.0(98.0, 216.0)
**TyG**	9.3 (8.8, 9.8)	9.1 (8.7, 9.7)	9.3 (8.8, 9.8)	9.1 (8.7, 9.6)	9.3 (8.8,9.9)	9.2 (8.7, 9.7)
**TyG_BMI**	305.7 (258.3, 355.6)	278.6 (240.8, 325.4)	301.6 (257.3, 353.1)	277.6 (237.4, 323.1)	301.9 (256.7, 357.6)	278.9 (240.9, 327.0)

Data are numeric (percentages), or median [interquartile spacing]. All estimates take into account the complex survey design. Q1: Quartile 1, Q2: Quartile 2, Q3: Quartile 3, Q4: Quartile 4, CVD: Cardiovascular disease, BMI: Body Mass Index, HbA1c: glycosylated hemoglobin, TC: Serum total cholesterol, FPG: Fasting Plasma Glucose, TG: Serum triglycerides, TyG: Triglyceride and glucose index, TyG_BMI: Triglyceride glucose-body mass index.

### Survival analysis and subgroup analyses

Supplementary Figure 1 emphasized the role of diet in diabetes prognosis, with Supplementary Figures 1B,E demonstrating that higher dietary scores in diabetic patients were associated with lower CV and all-cause mortality. After stratification, there was a gender-based interaction between HEI/AHEI and CV mortality (HEI: *p*-interaction = 0.006; AHEI: *p*-interaction = 0.003) (Table 2). Similarly, Table 3 further showed a significant gender-based interaction between HEI/AHEI/aMED index and all-cause mortality (HEI: *p*-interaction = 0.009; AHEI: *p*-interaction = 0.001; aMED index: *p*-interaction = 0.027). Interactions for other subgroups remained nonsignificant (*p*-interaction >0.05).

**Table 2:** Association of dietary indicators with CV mortality in different subgroups.

Variables	HEI		AHEI		aMED index	
	HR (95%CI)	P for interaction	HR (95%CI)	P for interaction	HR (95%CI)	P for interaction
Sex/gender		0.006		0.003		0.303
Male	0.91 (0.82-1.01)		0.81 (0.73-0.90)		0.98 (0.88-1.09)	
Female	1.15 (1.01-1.30)		1.03 (0.91-1.17)		1.07 (0.93-1.22)	
Age, years		0.171		0.288		0.114
<60	0.78 (0.62-0.98)		0.74 (0.59-0.93)		0.76 (0.60-0.96)	
≥60	0.92 (0.85-1.00)		0.84 (0.77-0.92)		0.93 (0.85-1.02)	
Race/ethnicity		0.397		0.836		0.938
Non-Hispanic Black	1.01 (0.85-1.19)		0.91 (0.77-1.08)		1.04 (0.87-1.24)	
Non-Hispanic White	1.04 (0.94-1.15)		0.90 (0.81-1.00)		1.01 (0.91-1.13)	
Mexican American	0.93 (0.73-1.18)		0.86 (0.68-1.08)		0.97 (0.75-1.25)	
Others	0.80 (0.58-1.09)		0.79 (0.59-1.07)		0.96 (0.70-1.31)	
Drinking status		0.629		0.413		0.482
Heavy	1.02 (0.87-1.19)		0.85 (0.73-0.99)		0.94 (0.80-1.11)	
Never/low/moderate	0.97 (0.89-1.07)		0.91 (0.83-1.00)		1.01 (0.92-1.12)	
Smoking status		0.123		0.217		0.896
Yes	1.08 (0.96-1.22)		0.95 (0.84-1.07)		1.02 (0.90-1.16)	
No	0.95 (0.85-1.06)		0.86 (0.77-0.96)		1.01 (0.90-1.13)	
Educational attainment		0.635		0.881		0.645
College and above	0.97 (0.85-1.12)		0.91 (0.79-1.04)		1.04 (0.90-1.20)	
Below College level	1.01 (0.92-1.12)		0.9 (0.82-0.99)		1.00 (0.90-1.11)	
Exercise status		0.56		0.306		0.946
Yes	1.03 (0.89-1.18)		0.95 (0.83-1.09)		1.01 (0.87-1.17)	
No	0.98 (0.89-1.08)		0.87 (0.79-0.96)		1.02 (0.92-1.13)	
Hypertension		0.147		0.908		0.274
Yes	0.95 (0.87-1.04)		0.88 (0.81-0.97)		0.97 (0.87-1.07)	
No	1.09 (0.93-1.27)		0.90 (0.77-1.04)		1.08 (0.92-1.27)	
HbA1c		0.129		0.068		0.238
<7.0	1.04 (0.94-1.16)		0.95 (0.85-1.05)		1.05 (0.94-1.17)	
≥7.0	0.92 (0.82-1.04)		0.81 (0.72-0.92)		0.95 (0.83-1.08)	

Data was expressed as HR (95% CI). The data was stratified by age, sex/gender, race/ethnicity, drinking status, smoking status, educational level, exercise status, hypertension status, and HbA1c level. Stratification variables themselves were not included in the model.

**Table 3:** Association of dietary indicators with all-cause mortality in different subgroups.

	HEI		AHEI		aMED index	
Variable	HR (95%CI)	P for interaction	HR (95%CI)	P for interaction	HR (95%CI)	P for interaction
Sex/gender		0.009		0.001		0.027
Male	0.97 (0.91-1.03)		0.89 (0.84-0.95)		1.00 (0.94-1.06)	
Female	1.09 (1.02-1.17)		1.04 (0.97-1.12)		1.11 (1.03-1.20)	
Age, years		0.326		0.199		0.099
<60	0.87 (0.77-0.99)		0.83 (0.74-0.93)		0.85 (0.75-0.96)	
≥60	0.93 (0.89-0.98)		0.90 (0.86-0.95)		0.96 (0.91-1.01)	
Race/ethnicity		0.06		0.292		0.093
Non-Hispanic Black	1.00 (0.90-1.10)		0.92 (0.83-1.02)		1.05 (0.94-1.17)	
Non-Hispanic White	1.06 (1.00-1.12)		0.98 (0.92-1.04)		1.08 (1.02-1.15)	
Mexican American	0.98 (0.87-1.10)		0.94 (0.83-1.06)		0.96 (0.85-1.10)	
Others	0.84 (0.72-1.00)		0.84 (0.72-0.98)		0.89 (0.75-1.05)	
Drinking status		0.095		0.364		0.355
Heavy	1.07 (0.98-1.17)		0.99 (0.91-1.07)		1.06 (0.97-1.16)	
Low/moderate/never	0.98 (0.93-1.03)		0.94 (0.89-1.00)		1.01 (0.96-1.07)	
Smoking status		0.06		0.431		0.303
Yes	1.09 (1.01-1.17)		0.98 (0.92-1.06)		1.09 (1.01-1.17)	
No	1.00 (0.94-1.06)		0.95 (0.90-1.01)		1.03 (0.97-1.10)	
Educational attainment		0.885		0.761		0.571
College and above	1.01 (0.93-1.10)		0.96 (0.89-1.04)		1.07 (0.98-1.16)	
Below College level	1.02 (0.97-1.08)		0.97 (0.92-1.03)		1.04 (0.98-1.10)	
Exercise status		0.469		0.251		0.983
Yes	1.04 (0.96-1.12)		1.00 (0.92-1.08)		1.05 (0.96-1.14)	
No	1.00 (0.95-1.06)		0.94 (0.89-1.00)		1.05 (0.99-1.12)	
Hypertension		0.67		0.933		0.836
Yes	1.00 (0.95-1.05)		0.95 (0.90-1.00)		1.03 (0.97-1.09)	
No	1.02 (0.94-1.11)		0.94 (0.87-1.03)		1.04 (0.95-1.14)	
HbA1c		0.663		0.385		0.807
<7.0	1.02 (0.96-1.08)		0.97 (0.91-1.03)		1.04 (0.98-1.11)	
≥7.0	1.00 (0.93-1.07)		0.93 (0.87-0.99)		1.03 (0.96-1.11)	

Data was expressed as HR (95% CI). The data was stratified by age, sex/gender, race/ethnicity, drinking status, smoking status, educational level, exercise status, hypertension status, and HbA1c level. Stratification variables themselves were not included in the model.

### Relationship between dietary scores and CV/all-cause mortality

After multivariate adjustment, in males, dietary scores were associated with CV mortality, all-cause mortality in an inverse relationship. The highest aMED index, HEI, and AHEI quartiles were significantly associated with reduced CV mortality (aMED index: HR = 0.58, 95% CI: 0.36–0.95, *p* = 0.027; HEI: HR = 0.46, 95% CI: 0.30–0.70, *p* < 0.001; AHEI: HR = 0.39, 95% CI: 0.25–0.62, *p* < 0.001) and reduced all-cause mortality (aMED index: HR = 0.75, 95% CI: 0.58–0.98, *p* = 0.03; AHEI: HR = 0.84, 95% CI: 0.64–1.10, *p* = 0.01) compared to the lowest quartiles of aMED index, HEI, and AHEI. However, among females, the associations between the dietary indices and CV mortality were not statistically significant, nor were the associations between the HEI, aMED index, and all-cause mortality, after similar adjustment. AHEI may be associated with reduced all-cause mortality in females (*p* = 0.012) (Tables 4, 5). Additionally, sensitivity analysis results further validated the accuracy of our results (Supplementary Tables 1, 2).

**Table 4:** Cardiovascular mortality in men and women with diabetes based on dietary index quartiles.

Cardiovascular mortality					
	Quartile 1	Quartile 2	Quartile 3	Quartile 4	*p* for trend
HEI					
Male					
Model 1	1	0.99 (0.66–1.46)	0.73 (0.47–1.12)	0.42 (0.28–0.61)	<0.001
Model 2	1	1.06 (0.70–1.61)	0.82 (0.54–1.23)	0.47 (0.32–0.69)	<0.001
Model 3	1	1.05 (0.69–1.61)	0.80 (0.52–1.23)	0.46 (0.30–0.70)	<0.001
Female					
Model 1	1	0.91 (0.57–1.47)	0.89 (0.57–1.40)	0.77 (0.50–1.19)	0.677
Model 2	1	0.97 (0.60–1.59)	0.98 (0.62–1.54)	0.95 (0.58–1.57)	0.758
Model 3	1	1.02 (0.62–1.70)	1.02 (0.65–1.59)	1.03 (0.60–1.75)	0.608
AHEI					
Male					
Model 1	1	0.75 (0.52–1.09)	0.46 (0.32–0.68)	0.29 (0.19–0.45)	<0.001
Model 2	1	0.93 (0.64–1.34)	0.56 (0.37–0.85)	0.39 (0.25–0.60)	<0.001
Model 3	1	0.92 (0.63–1.34)	0.56 (0.36–0.86)	0.39 (0.25–0.62)	<0.001
Female					
Model 1	1	1.01 (0.61–1.65)	0.58 (0.36–0.94)	0.67 (0.43–1.04)	0.002
Model 2	1	1.05 (0.62–1.76)	0.59 (0.36–0.97)	0.82 (0.51–1.34)	0.042
Model 3	1	1.04 (0.64–1.71)	0.58 (0.35–0.95)	0.86 (0.52–1.40)	0.064
aMED index					
Male					
Model 1	1	0.85 (0.54–1.34)	0.60 (0.36–1.01)	0.51 (0.33–0.80)	0.003
Model 2	1	0.85 (0.54–1.35)	0.62 (0.37–1.04)	0.56 (0.36–0.86)	0.014
Model 3	1	0.90 (0.56–1.46)	0.63 (0.36–1.11)	0.58 (0.36–0.95)	0.027
Female					
Model 1	1	0.53 (0.32–0.89)	0.49 (0.25–0.98)	0.53 (0.30–0.92)	0.425
Model 2	1	0.63 (0.38–1.04)	0.61 (0.30–1.22)	0.66 (0.34–1.26)	0.928
Model 3	1	0.65 (0.40–1.08)	0.63 (0.32–1.27)	0.71 (0.36–1.40)	0.877

Data was expressed as HR (95% CI). Model 1: adjusted for demographic factors (age, race/ethnicity). Model 2: Includes additional adjustments for socio-behavioral factors (educational level, drinking patterns, smoking status, and exercise level), baseline hypertension disease status, and baseline cardiovascular disease status. Model 3: Further adjustments were made for metabolic indicators and health status affecting cardiovascular risk (HbA1c, BMI, cholesterol, blood glucose, triglycerides), as well as for novel indicators such as the triglyceride-glucose (TyG) index and its body mass index-adjusted variant (TyG-BMI index).

**Table 5:** All-cause mortality in men and women with diabetes based on dietary index quartiles.

All-cause mortality					
	Quartile 1	Quartile 2	Quartile 3	Quartile 4	*p* for trend
HEI					
Male					
Model 1	1	0.95 (0.74–1.20)	1.01 (0.78–1.32)	0.62 (0.48–0.78)	0.001
Model 2	1	1.03 (0.80–1.33)	1.22 (0.93–1.59)	0.75 (0.58–0.96)	0.062
Model 3	1	1.03 (0.79–1.34)	1.22 (0.93–1.59)	0.76 (0.59–0.99)	0.103
Female					
Model 1	1	0.81 (0.63–1.05)	0.83 (0.64–1.08)	0.66 (0.50–0.87)	0.011
Model 2	1	0.89 (0.68–1.15)	0.93 (0.71–1.21)	0.81 (0.61–1.08)	0.238
Model 3	1	0.90 (0.69–1.19)	0.96 (0.73–1.26)	0.80 (0.60–1.07)	0.147
AHEI					
Male					
Model 1	1	0.98 (0.77–1.24)	0.73 (0.59–0.90)	0.58 (0.45–0.75)	<0.001
Model 2	1	1.19 (0.95–1.50)	0.87 (0.70–1.10)	0.79 (0.61–1.03)	0.002
Model 3	1	1.21 (0.96–1.54)	0.90 (0.71–1.12)	0.84 (0.64–1.10)	0.01
Female					
Model 1	1	1.11 (0.83–1.48)	0.77 (0.57–1.04)	0.75 (0.58–0.97)	<0.001
Model 2	1	1.16 (0.86–1.58)	0.82 (0.60–1.12)	0.92 (0.69–1.22)	0.025
Model 3	1	1.13 (0.84–1.53)	0.82 (0.60–1.11)	0.89 (0.67–1.18)	0.012
aMED index					
Male					
Model 1	1	0.94 (0.72–1.24)	0.95 (0.72–1.26)	0.64 (0.49–0.84)	<0.001
Model 2	1	0.96 (0.73–1.27)	0.98 (0.74–1.30)	0.72 (0.56–0.93)	0.011
Model 3	1	1.00 (0.76–1.33)	1.01 (0.75–1.37)	0.75 (0.58–0.98)	0.03
Female					
Model 1	1	0.79 (0.57–1.11)	0.65 (0.44–0.96)	0.72 (0.52–1.01)	0.172
Model 2	1	0.90 (0.64–1.28)	0.79 (0.52–1.19)	0.91 (0.63–1.32)	0.941
Model 3	1	0.90 (0.63–1.27)	0.81 (0.54–1.21)	0.89 (0.62–1.30)	0.898

Data was expressed as HR (95% CI). Model 1: adjusted for demographic factors (age, race/ethnicity). Model 2: Includes additional adjustments for socio-behavioral factors (educational level, drinking patterns, smoking status, and exercise level), baseline hypertension disease status, and baseline cardiovascular disease status. Model 3: Further adjustments were made for metabolic indicators and health status affecting cardiovascular risk (HbA1c, BMI, cholesterol, blood glucose, triglycerides), as well as for novel indicators such as the triglyceride-glucose (TyG) index and its body mass index-adjusted variant (TyG-BMI index).

### Dose–response relationship

**Figure 2:**
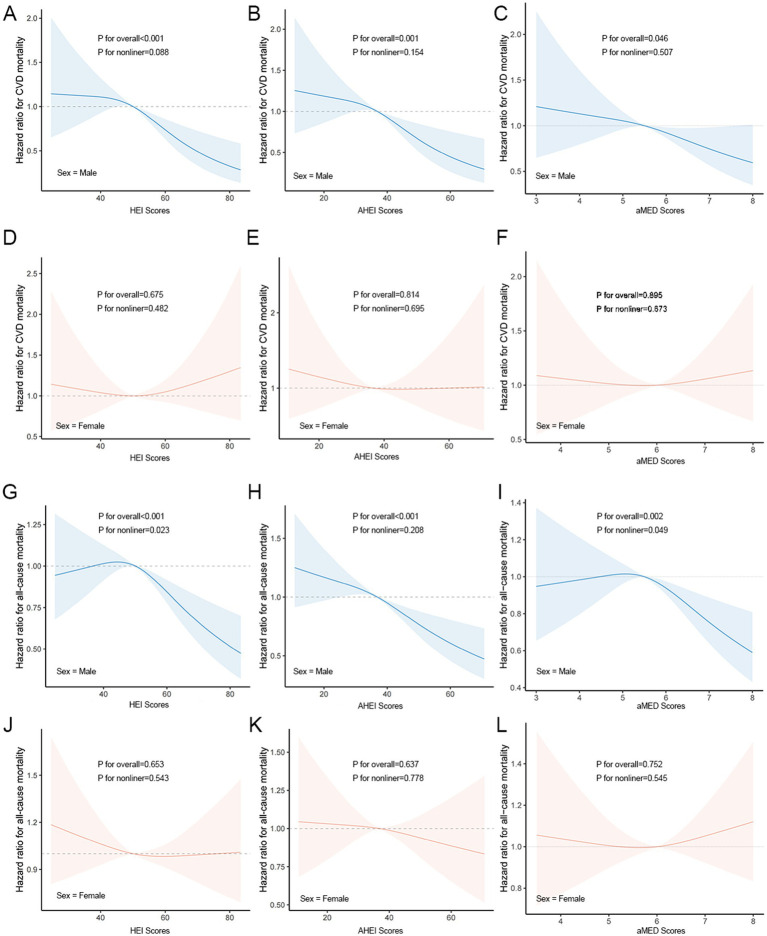
Dose–response relationships between dietary indicators and CV/all-cause mortality. **(A–C)** Associations between HEI **(A)**, AHEI **(B)**, and aMED **(C)** scores and CV mortality in males. **(D–F)** Associations between HEI **(D)**, AHEI **(E)**, and aMED **(F)** scores and CV mortality in females. **(G–I)** Associations between HEI **(G)**, AHEI **(H)**, and aMED **(I)** scores and all-cause mortality in males. **(J–L)** Associations between HEI **(J)**, AHEI **(K)**, and aMED **(L)** scores and all-cause mortality in females.

Higher dietary scores were associated with a lower CV/all-cause mortality in males, whereas in females there was no significant change. Among males, there was a significant linear relationship between the CV mortality and the three dietary indices (*p* nonlinear >0.05; *p* overall <0.05) (Figures 2A–C). In females, HEI, AHEI, and aMED scores were not significantly associated with CV mortality, with no clear linear or nonlinear dose-response relationships observed (Figures 2D–F). There was a linear relationship between the all-cause mortality and the AHEI (*p* nonlinear >0.05; *p* overall<0.05) (Figure 2H) and a significant non-linear relationship with the HEI, aMED index (*p* nonlinear <0.05; *p* overall<0.05) (Figures 2G,I).

### Association between components of aMED and CV/all-cause mortality

**Figure 3:**
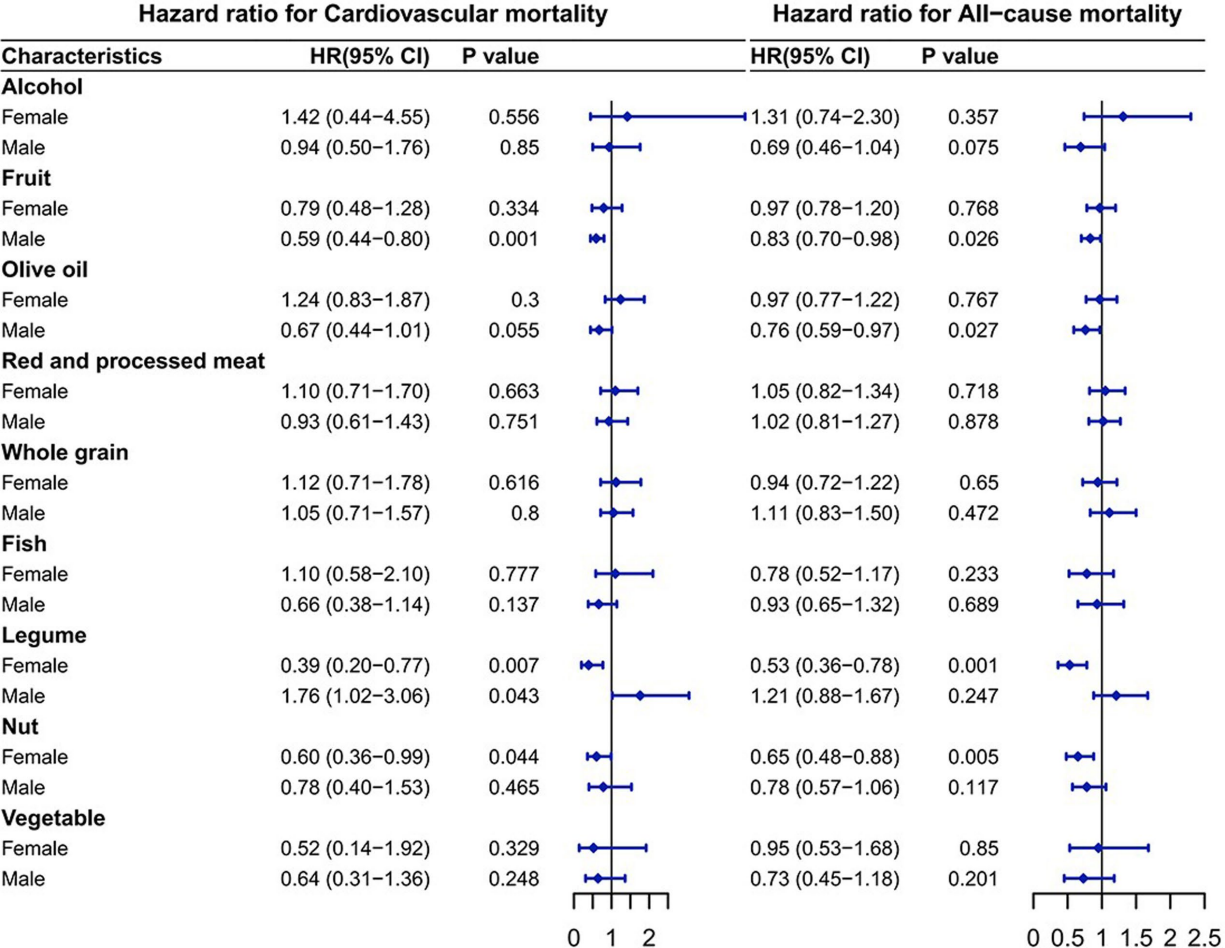
Association of the components of the alternative Mediterranean diet with mortality. The model was adjusted based on age, race/ethnicity, educational level, drinking patterns, smoking status, exercise level, baseline hypertension status, baseline cardiovascular disease status, HbA1c level, BMI, cholesterol, glucose, triglycerides, TyG and TyG-BMI.

As shown in Figure 3, among the components, legume intake was significantly connected to higher CV mortality in males, whereas fruit and olive oil intake were significantly connected to lower mortality. Legume and nut intake was significantly connected to lower mortality in females. Using the composition of the alternative Mediterranean diet as an example, the HRs for the CV mortality was as follows: in males, the HR for fruits was 0.59 (95% CI: 0.44–0.80; *p* = 0.001), and the HR for legumes was 1.76 (95% CI: 1.02–3.06; *p* = 0.043). In females, the HR for legumes was 0.39 (95% CI: 0.20–0.77; *p* = 0.007) and for nuts was 0.60 (95% CI: 0.36–0.99; *p* = 0.044). The HRs for the all-cause mortality from T2DM corresponding to additional daily intake were 0.83 (95% CI: 0.70–0.98; *p* = 0.026) for fruits and 0.76 (95% CI: 0.59–0.97; *p* = 0.027) for olive oil in males. In females, the HR was 0.53 (95% CI: 0.36–0.78; *p* = 0.001) for legumes and 0.65 (95% CI: 0.48–0.88; *p* = 0.005) for nuts.

## Discussion

Our study provided the detailed report on the impact of gender differences on diet and long-term mortality in a T2DM population. Previous cohort studies have demonstrated a 32% lower risk of CVD mortality and an 18% lower risk of all-cause mortality in participants in the highest quintile of the HEI-2015 compared to those in the lowest quintile of the HEI-2015, after controlling for potential confounders (22). In a follow-up investigation over a median follow-up time of 6.9 years, subjects in the highest quintile of the AHEI had a 27% lower risk of CVD mortality and a 16% lower risk of all-cause mortality, underscoring the significant impact of dietary habits on physical health (23). Notably, higher dietary scores were significantly associated with lower CV/all-cause mortality in males. However, there was no significant association between these dietary indices and the prognosis of females with diabetes (Tables 4, 5). This gender disparity may be attributable to several factors. First, adherence to dietary guidelines tends to differ significantly between genders, as indicated by previous studies. A German study, for instance, reported marked gender differences in dietary adherence and glycaemic control, suggesting that males might cope more effectively with dietary recommendations compared to females, who often adopt independent management strategies (24). Furthermore, female patients have to cook and care for their families, which makes it difficult for them to follow their own diets and eating schedules. Second, physiological differences related to reproductive factors may place females with diabetes at greater cardiometabolic risk. Females typically possess less fat-free muscle mass compared to males, potentially exacerbating insulin resistance and dyslipidaemia (25). A decline in estrogen after menopause may also be a potential reason for females’ poorer prognosis (26). These findings strongly indicate that, as with virtually all medical conditions, gender should be considered in the personalized treatment of diabetes.

Recent studies have demonstrated that the lipid profiles of diabetic females are less favorable than those of males, suggesting that gender differences may influence dietary habits (27). Epidemiological evidence currently supports the existence of gender differences in responses to the Mediterranean diet, which significantly enhances insulin homeostasis in males, with no comparable effects observed in females (28). Females also exhibit a reduced response to alterations in dietary fat and carbohydrate intake compared to males (29). Experimental and clinical studies further illustrate gender differences in substrate utilization, with females predominantly converting non-esterified fatty acids (NEFAs) into triglycerides during resting and postprandial states, favoring lipid storage, whereas males typically oxidize NEFAs for immediate energy generation (25, 30). These metabolic distinctions offer critical insights for tailoring dietary interventions to optimize gender-specific outcomes in diabetes management.

There are substantial evidences that dietary components potentially influence the risk of diabetes in genetically predisposed individuals by regulating inflammation and oxidation processes (31). For example, n-3 fatty acids and fiber- and phytochemical-rich plant-based foods (whole grains, fruits, vegetables, and legumes) possess well-established anti-inflammatory properties, reducing inflammation through antioxidant activity, interference with oxidative stress signaling, and inhibition of pro-inflammatory signal transduction (32). Intake of these diets can effectively improve endothelial dysfunction and reduce the risk of cardiovascular death in patients with diabetes.

Legumes, central components of traditional plant-based diets globally, contain abundant proteins, fibers, magnesium, and bioactive polyphenols (33). Epidemiological analyses involving nearly 100,000 females and over 56,000 males have indicated gender-dependent associations between legume consumption and diabetes prevalence, with legume intake significantly reducing diabetes risk in females but presenting inverse associations in males (34). Notably, the findings of this study suggested that legumes intake has a protective effect on females with diabetes, significantly reducing the CV/all-cause mortality. However, in males, legumes intake significantly increased the CV mortality (Figure 3). This finding demonstrates the prognostic value of legumes in females with T2DM and their positive contribution to females’ health. The protective function of legumes on females with diabetes possibly be due to a variety of biological reasons. The most likely explanation has to do with isoflavones, a type of phytoestrogens found almost exclusively in soybeans and other legumes (35). Estrogen is known to be a potential heart protector and an immunomodulator of inflammatory responses in atherosclerosis (36). Because the chemical structure of isoflavones is similar to estrogen, isoflavones can mimic the effects of estrogen on the human body (37). When males ingest legumes, only about 30 percent produce equol, a metabolite of legumes that is an important isoflavone for humans (38, 39). Polyphenols, including lignans and ketones, also exhibit potent antioxidant properties, potentially contributing to protective effects against diabetes progression (40). Our results also suggested that fruit intake significantly reduced CV/all-cause mortality in males with diabetes (Figure 3). Fruits contain heart-protective components like fiber, folate, nitrates, vitamins, and non-nutritive phytochemicals, including flavonoids such as anthocyanins, flavonols, and flavones, which are abundant in common fruits like berries, citrus fruits, apples, and grapes (41). The results of a randomized controlled trial suggest that flavonoid-rich fruits improve microvascular reactivity and inflammatory status in males (42). Flavonoids have been reported to promote vasodilation and improve vascular function by activating eNOS or enhancing the availability of endogenous NO (43).

A key strength of the study is its use of NHANES data, a large, nationally representative survey known for its timeliness and high quality. Secondly, we made multivariate adjustments: The study accounted for multiple potential confounders in the analysis, including new metabolic markers (TyG and TyG-BMI) that affect cardiovascular risk.

### Limitations

Firstly, as an observational study, we cannot draw definitive causal conclusions about the relationship between dietary scores and mortality. Residual confounding from unmeasured or inadequately adjusted factors may influence the associations observed. Secondly, dietary assessments and covariates relied upon self-reported recall, potentially introducing recall bias. Moreover, dietary quality scores were measured only at baseline, limiting insights into the impact of dietary changes during follow-up on long-term mortality outcomes. Future longitudinal studies addressing these limitations will be essential to confirm our findings.

## Conclusion

There are gender differences in the relationship between diabetes patients’ diet and their risk of CV and all-cause deaths. Higher dietary scores are significantly associated with CV/all-cause mortality in males but not in females. Legumes intake is unfavorable for males with diabetes, but was substantially connected to lower CV/all-cause mortality in females, with a protective effect in females with diabetes.

## Data Availability

The original contributions presented in the study are included in the article/Supplementary material, further inquiries can be directed to the corresponding authors.
