# Supplementary material for: Sex-specific associations between diet quality and mortality in adults with diabetes: findings from NHANES 2001-2018
Source: Front Nutr. 2025 Apr 16;12:1576983. doi: 10.3389/fnut.2025.1576983 (PMC12040670; doi:10.3389/fnut.2025.1576983)
Supplement: Supplementary file 1 [file Table_1.docx]

**Supplementary Table 1 Association between dietary indices and CV/all-cause mortality in T2DM (excluding those with cardiovascular disease)**

|  | Q1 | Q2 | Q3 | Q4 | P trend |
| --- | --- | --- | --- | --- | --- |
| Cardiovascular mortality | | | | | |
|  | HEI | | | |  |
| Male |  |  |  |  |  |
| Model 1 | 1.00 | 0.84 (0.44-1.62) | 0.72 (0.39-1.34) | 0.37 (0.22-0.65) | <0.001 |
| Model 2 | 1.00 | 0.83 (0.42-1.63) | 0.82 (0.43-1.58) | 0.41 (0.22-0.75) | 0.005 |
| Model 3 | 1.00 | 0.82 (0.41-1.64) | 0.78 (0.4-1.53) | 0.42 (0.23-0.76) | 0.008 |
| Female |  |  |  |  |  |
| Model 1 | 1.00 | 0.93 (0.52-1.67) | 0.86 (0.44-1.69) | 1.01 (0.53-1.91) | 0.546 |
| Model 2 | 1.00 | 1.05 (0.56-1.94) | 1.09 (0.55-2.17) | 1.25 (0.61-2.56) | 0.367 |
| Model 3 | 1.00 | 1.09 (0.57-2.08) | 1.1 (0.56-2.19) | 1.23 (0.59-2.56) | 0.517 |
|  | AHEI | | | |  |
| Male |  |  |  |  |  |
| Model 1 | 1.00 | 0.68 (0.38-1.23) | 0.48 (0.25-0.91) | 0.31 (0.17-0.56) | <0.001 |
| Model 2 | 1.00 | 0.76 (0.42-1.37) | 0.5 (0.26-0.94) | 0.38 (0.2-0.72) | 0.001 |
| Model 3 | 1.00 | 0.76 (0.42-1.38) | 0.47 (0.24-0.94) | 0.39 (0.21-0.75) | 0.002 |
| Female |  |  |  |  |  |
| Model 1 | 1.00 | 1.27 (0.67-2.42) | 0.75 (0.4-1.42) | 1.11 (0.62-2.01) | 0.460 |
| Model 2 | 1.00 | 1.13 (0.56-2.27) | 0.84 (0.42-1.66) | 1.19 (0.63-2.26) | 0.749 |
| Model 3 | 1.00 | 1.03 (0.51-2.06) | 0.81 (0.41-1.6) | 1.06 (0.56-1.99) | 0.561 |
|  | aMED index | | | |  |
| Male |  |  |  |  |  |
| Model 1 | 1.00 | 0.45 (0.21-0.97) | 0.47 (0.24-0.94) | 0.37 (0.2-0.69) | 0.003 |
| **Supplementary Table 1 Association between dietary indices and CV/all-cause mortality in T2DM (excluding those with cardiovascular disease) (continue)** | | | | | |
| Model 2 | 1.00 | 0.46 (0.22-0.98) | 0.5 (0.25-0.99) | 0.45 (0.24-0.83) | 0.023 |
| Model 3 | 1.00 | 0.45 (0.21-0.94) | 0.5 (0.25-0.98) | 0.47 (0.24-0.89) | 0.047 |
| Female |  |  |  |  |  |
| Model 1 | 1.00 | 0.37 (0.18-0.74) | 0.42 (0.18-0.97) | 0.59 (0.28-1.27) | 0.753 |
| Model 2 | 1.00 | 0.46 (0.21-0.99) | 0.57 (0.22-1.47) | 0.85 (0.35-2.04) | 0.346 |
| Model 3 | 1.00 | 0.47 (0.21-1.04) | 0.56 (0.21-1.53) | 0.82 (0.32-2.16) | 0.444 |
| All-cause mortality | | | | | |
|  | HEI | | | |  |
| Male |  |  |  |  |  |
| Model 1 | 1.00 | 0.81 (0.57-1.16) | 0.96 (0.67-1.37) | 0.57 (0.4-0.8) | 0.01 |
| Model 2 | 1.00 | 0.83 (0.58-1.17) | 1.14 (0.77-1.68) | 0.67 (0.45-0.99) | 0.211 |
| Model 3 | 1.00 | 0.82 (0.57-1.17) | 1.15 (0.78-1.7) | 0.68 (0.47-1) | 0.270 |
| Female |  |  |  |  |  |
| Model 1 | 1.00 | 0.87 (0.59-1.29) | 0.75 (0.52-1.07) | 0.76 (0.53-1.1) | 0.190 |
| Model 2 | 1.00 | 0.96 (0.66-1.4) | 0.9 (0.64-1.27) | 0.93 (0.66-1.33) | 0.780 |
| Model 3 | 1.00 | 1.04 (0.7-1.55) | 0.96 (0.68-1.35) | 0.93 (0.66-1.32) | 0.504 |
|  | AHEI | | | |  |
| Male |  |  |  |  |  |
| Model 1 | 1.00 | 1 (0.73-1.38) | 0.75 (0.56-1) | 0.55 (0.38-0.79) | <0.001 |
| Model 2 | 1.00 | 1.16 (0.83-1.64) | 0.83 (0.61-1.12) | 0.72 (0.48-1.07) | <0.001 |
| Model 3 | 1.00 | 1.18 (0.84-1.68) | 0.86 (0.62-1.19) | 0.78 (0.52-1.15) | 0.004 |
| Female |  |  |  |  |  |
| Model 1 | 1.00 | 1.24 (0.87-1.78) | 0.83 (0.56-1.24) | 0.92 (0.65-1.3) | 0.054 |
| **Supplementary Table 1 Association between dietary indices and CV/all-cause mortality in T2DM (excluding those with cardiovascular disease) (continue)** | | | | | |
| Model 2 | 1.00 | 1.3 (0.9-1.89) | 0.95 (0.63-1.43) | 1.07 (0.74-1.56) | 0.347 |
| Model 3 | 1.00 | 1.25 (0.87-1.79) | 0.96 (0.64-1.44) | 1.02 (0.7-1.46) | 0.219 |
|  | aMED index | | | |  |
| Male |  |  |  |  |  |
| Model 1 | 1.00 | 0.76 (0.51-1.13) | 0.88 (0.61-1.25) | 0.55 (0.38-0.79) | 0.001 |
| Model 2 | 1.00 | 0.77 (0.53-1.11) | 0.91 (0.63-1.32) | 0.66 (0.46-0.95) | 0.005 |
| Model 3 | 1.00 | 0.79 (0.53-1.16) | 0.93 (0.63-1.37) | 0.68 (0.48-0.98) | 0.03 |
| Female |  |  |  |  |  |
| Model 1 | 1.00 | 0.86 (0.54-1.38) | 0.58 (0.35-0.99) | 0.82 (0.53-1.29) | 0.590 |
| Model 2 | 1.00 | 0.96 (0.58-1.6) | 0.74 (0.43-1.26) | 1.06 (0.66-1.71) | 0.457 |
| Model 3 | 1.00 | 0.97 (0.58-1.61) | 0.76 (0.44-1.29) | 1.05 (0.64-1.72) | 0.587 |

Data was expressed as HR (95% CI). Model 1: adjusted for demographic factors (age, race/ethnicity). Model 2: Includes additional adjustments for socio-behavioral factors (educational level, drinking patterns, smoking status, and exercise level), baseline hypertension disease status, and baseline cardiovascular disease status. Model 3: Further adjustments were made for metabolic indicators and health status affecting cardiovascular risk (HbA1c, BMI, cholesterol, blood glucose, triglycerides), as well as for novel indicators such as the triglyceride-glucose (TyG) index and its body mass index-adjusted variant (TyG-BMI index).
