# Supplementary material for: Sex-specific associations between diet quality and mortality in adults with diabetes: findings from NHANES 2001-2018
Source: Front Nutr. 2025 Apr 16;12:1576983. doi: 10.3389/fnut.2025.1576983 (PMC12040670; doi:10.3389/fnut.2025.1576983)
Supplement: Supplementary file 2 [file Table_2.docx]

**Supplementary Table 2 Association between dietary indices and CV/all-cause mortality in T2DM after excluding participants who died within 2 years of follow-up**

|  | Q1 | Q2 | Q3 | Q4 | P trend |
| --- | --- | --- | --- | --- | --- |
| Cardiovascular mortality | | | | | |
|  | HEI | | | |  |
| Male |  |  |  |  |  |
| Model 1 | 1.00 | 0.91 (0.6-1.38) | 0.92 (0.6-1.41) | 0.38 (0.24-0.59) | <0.001 |
| Model 2 | 1.00 | 0.94 (0.61-1.46) | 1.03 (0.67-1.58) | 0.43 (0.27-0.68) | <0.001 |
| Model 3 | 1.00 | 0.96 (0.61-1.5) | 1.05 (0.68-1.61) | 0.45 (0.29-0.72) | 0.001 |
| Female |  |  |  |  |  |
| Model 1 | 1.00 | 1.01 (0.61-1.65) | 0.83 (0.51-1.37) | 0.75 (0.49-1.14) | 0.422 |
| Model 2 | 1.00 | 1.09 (0.66-1.82) | 1 (0.62-1.63) | 0.94 (0.59-1.5) | 0.949 |
| Model 3 | 1.00 | 1.12 (0.67-1.88) | 1.02 (0.64-1.65) | 0.96 (0.6-1.55) | 0.926 |
|  | AHEI | | | |  |
| Male |  |  |  |  |  |
| Model 1 | 1.00 | 0.82 (0.55-1.22) | 0.45 (0.29-0.67) | 0.31 (0.2-0.49) | <0.001 |
| Model 2 | 1.00 | 0.94 (0.63-1.38) | 0.49 (0.32-0.75) | 0.38 (0.24-0.61) | <0.001 |
| Model 3 | 1.00 | 0.96 (0.65-1.42) | 0.5 (0.33-0.76) | 0.41 (0.25-0.65) | <0.001 |
| Female |  |  |  |  |  |
| Model 1 | 1.00 | 0.98 (0.58-1.67) | 0.51 (0.32-0.82) | 0.66 (0.42-1.05) | 0.002 |
| Model 2 | 1.00 | 0.96 (0.53-1.74) | 0.56 (0.34-0.91) | 0.75 (0.45-1.26) | 0.027 |
| Model 3 | 1.00 | 0.94 (0.53-1.68) | 0.55 (0.33-0.89) | 0.75 (0.44-1.27) | 0.030 |
|  | aMED index | | | |  |
| Male |  |  |  |  |  |
| Model 1 | 1.00 | 0.85 (0.48-1.5) | 0.66 (0.4-1.07) | 0.47 (0.29-0.76) | 0.001 |
| Model 2 | 1.00 | 0.84 (0.47-1.49) | 0.68 (0.42-1.1) | 0.53 (0.33-0.85) | 0.015 |
| **Supplementary Table 2 Association between dietary indices and CV/all-cause mortality in T2DM after excluding participants who died within 2 years of follow-up (continue)** | | | | | |
| Model 3 | 1.00 | 0.92 (0.5-1.7) | 0.72 (0.43-1.18) | 0.59 (0.36-0.98) | 0.038 |
| Female |  |  |  |  |  |
| Model 1 | 1.00 | 0.54 (0.31-0.93) | 0.51 (0.25-1.02) | 0.51 (0.28-0.93) | 0.383 |
| Model 2 | 1.00 | 0.61 (0.35-1.06) | 0.62 (0.3-1.31) | 0.68 (0.34-1.34) | 0.982 |
| Model 3 | 1.00 | 0.64 (0.37-1.09) | 0.64 (0.3-1.35) | 0.71 (0.35-1.42) | 0.913 |
| All-cause mortality | | | | | |
|  | HEI | | | |  |
| Male |  |  |  |  |  |
| Model 1 | 1.00 | 1.01 (0.8-1.27) | 1.09 (0.84-1.42) | 0.61 (0.48-0.77) | 0.002 |
| Model 2 | 1.00 | 1.06 (0.83-1.36) | 1.29 (0.98-1.71) | 0.74 (0.56-0.96) | 0.202 |
| Model 3 | 1.00 | 1.07 (0.83-1.38) | 1.3 (0.99-1.72) | 0.76 (0.58-0.99) | 0.310 |
| Female |  |  |  |  |  |
| Model 1 | 1.00 | 0.94 (0.72-1.21) | 0.82 (0.61-1.11) | 0.73 (0.55-0.97) | 0.017 |
| Model 2 | 1.00 | 1.04 (0.81-1.34) | 0.98 (0.73-1.31) | 0.91 (0.69-1.2) | 0.320 |
| Model 3 | 1.00 | 1.08 (0.83-1.41) | 1 (0.75-1.35) | 0.89 (0.68-1.18) | 0.164 |
|  | AHEI | | | |  |
| Male |  |  |  |  |  |
| Model 1 | 1.00 | 1.01 (0.77-1.32) | 0.76 (0.62-0.94) | 0.6 (0.45-0.79) | <0.001 |
| Model 2 | 1.00 | 1.2 (0.91-1.58) | 0.88 (0.7-1.1) | 0.78 (0.57-1.07) | 0.007 |
| Model 3 | 1.00 | 1.23 (0.92-1.63) | 0.89 (0.71-1.12) | 0.83 (0.61-1.14) | 0.027 |
| Female |  |  |  |  |  |
| Model 1 | 1.00 | 1.05 (0.77-1.44) | 0.74 (0.55-1) | 0.76 (0.58-0.99) | <0.001 |
| Model 2 | 1.00 | 1.09 (0.78-1.53) | 0.82 (0.59-1.13) | 0.88 (0.66-1.18) | 0.001 |
| Model 3 | 1.00 | 1.06 (0.75-1.51) | 0.82 (0.6-1.12) | 0.85 (0.64-1.14) | 0.005 |
| **Supplementary Table 2 Association between dietary indices and CV/all-cause mortality in T2DM after excluding participants who died within 2 years of follow-up (continue)** | | | | | |
|  | aMED index | | | |  |
| Male |  |  |  |  |  |
| Model 1 | 1.00 | 0.94 (0.67-1.34) | 0.94 (0.69-1.26) | 0.62 (0.46-0.83) | <0.001 |
| Model 2 | 1.00 | 0.93 (0.66-1.31) | 0.97 (0.72-1.3) | 0.72 (0.54-0.96) | 0.021 |
| Model 3 | 1.00 | 0.97 (0.68-1.39) | 1 (0.73-1.36) | 0.75 (0.56-1.02) | 0.044 |
| Female |  |  |  |  |  |
| Model 1 | 1.00 | 0.8 (0.56-1.15) | 0.69 (0.46-1.03) | 0.75 (0.52-1.07) | 0.301 |
| Model 2 | 1.00 | 0.89 (0.61-1.29) | 0.83 (0.54-1.28) | 0.96 (0.65-1.42) | 0.576 |
| Model 3 | 1.00 | 0.88 (0.6-1.27) | 0.84 (0.55-1.28) | 0.93 (0.63-1.38) | 0.769 |

Data was expressed as HR (95% CI). Model 1: adjusted for demographic factors (age, race/ethnicity). Model 2: Includes additional adjustments for socio-behavioral factors (educational level, drinking patterns, smoking status, and exercise level), baseline hypertension disease status, and baseline cardiovascular disease status. Model 3: Further adjustments were made for metabolic indicators and health status affecting cardiovascular risk (HbA1c, BMI, cholesterol, blood glucose, triglycerides), as well as for novel indicators such as the triglyceride-glucose (TyG) index and its body mass index-adjusted variant (TyG-BMI index).
