# Supplementary figures and images for: Sex-specific associations between diet quality and mortality in adults with diabetes: findings from NHANES 2001-2018
Source: Front Nutr. 2025 Apr 16;12:1576983. doi: 10.3389/fnut.2025.1576983 (PMC12040670; doi:10.3389/fnut.2025.1576983)

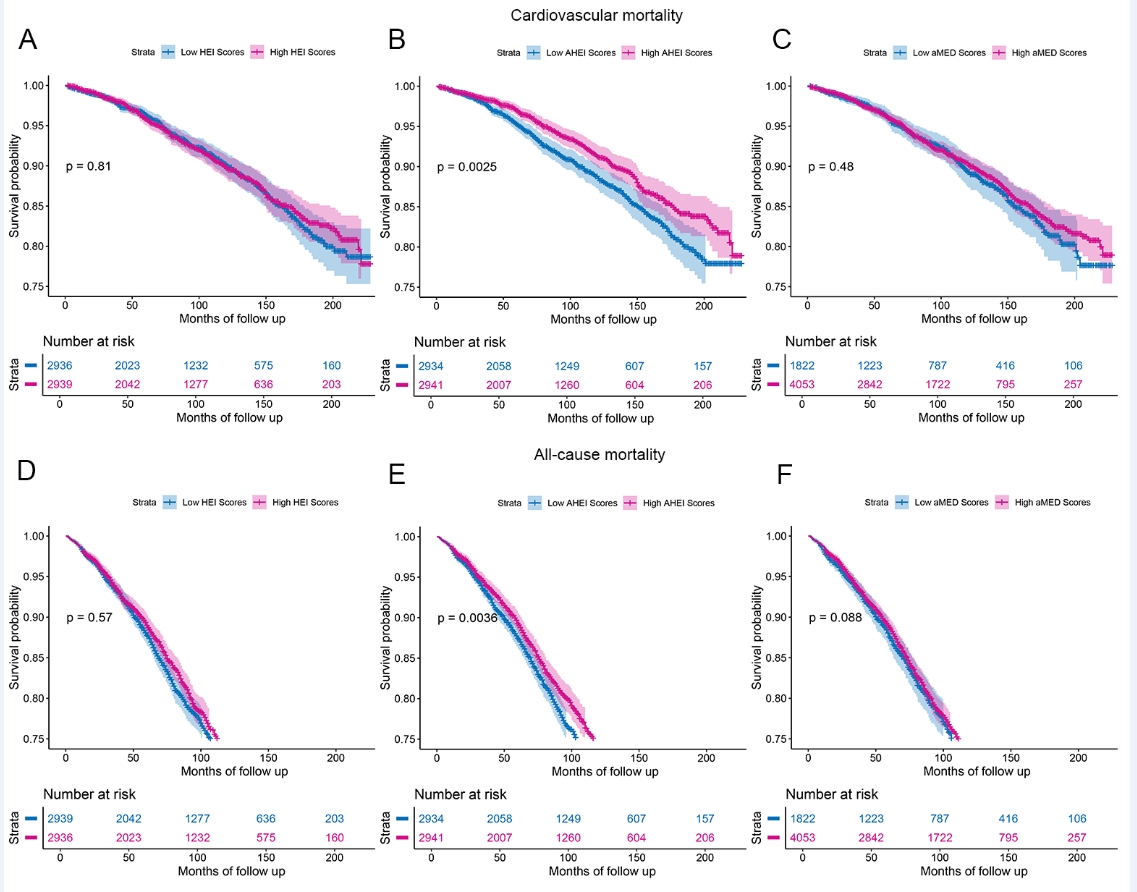

Supplement: Supplementary file 3 [file Image_1.jpg]
